# Classification of Patients with Dementia with Lewy Bodies by Event‐related Oscillations

**DOI:** 10.1002/alz.084655

**Published:** 2025-01-09

**Authors:** Bahar Güntekin, Rümeysa Duygun, Burcu Bölükbaş, Hikmet Buse Kuloğlu, İrem Yemeniciler, Harun Yirikogullari, Nesrin Helvacı‐Yılmaz, Lutfu Hanoğlu

**Affiliations:** ^1^ Istanbul Medipol University, Istanbul Turkey

## Abstract

**Background:**

In the EEG research of Alzheimer's Disease (AD) and other diseases belonging to dementia, the literature is rapidly growing to indicate biomarkers specific to the type of dementia. The literature showed firm conclusions that decreasing event‐related delta and theta responses could be a biomarker showing cognitive decline (Güntekin et al. 2022). However, event‐related EEG biomarkers of Lewy Body with dementia (DLB) were not studied well. The present study aims to differentiate the event‐related oscillations between DLB patients with healthy controls during cognitive paradigms.

**Method:**

19 DLB patients and aged‐matched 20 healthy controls (HC) were included in the study. 32‐channel EEG was recorded during the visual oddball paradigm. Event‐related power spectrum and phase‐locking were analyzed for the delta (0,5‐3,5 Hz), theta (4‐7 Hz), alpha (8‐13 Hz), beta (15‐28 Hz), and gamma (28‐48 Hz) frequency bands. The statistical analyses were run with repeated measures of ANOVA.

**Result:**

DLB patients had decreased event‐related delta and theta power and phase‐locking compared to HC (p<0.001; p<0.001). There is no differentiation in event‐related alpha, beta, and gamma power between DLB patients and HC controls. The phase locking factor could differentiate better between healthy controls and DLB patients in the higher frequency bands. Event‐related alpha (p=0.002), beta(p=0.001), and gamma (p=0.010) phase locking of DLB patients were decreased in DLB patients compared to HC.

**Conclusion:**

The present study showed that, like other types of dementia, DLB patients had reduced delta and theta power and phase locking compared to HC. The event‐related phase locking factor in all frequency bands was more effective in differentiating DLB than HC and could be used a biomarker. Furthermore, the phase locking factor in delta, theta, alpha, beta, and gamma frequency bands could be used with machine learning approaches to classify HC and DLB.

